# Oral cancer screening in the Bangladeshi community of Tower Hamlets: a social model

**DOI:** 10.1038/sj.bjc.6605394

**Published:** 2009-12-03

**Authors:** H Nunn, A Lalli, F Fortune, R Croucher

**Affiliations:** 1 Health Information Department, Cancer Research UK, 61 Lincoln's Inn Fields, London WC2A 3PX, UK; 2 Centre for Clinical and Diagnostic Oral Sciences, Institute of Dentistry, Barts and the London School of Medicine and Dentistry, Queen Mary, University of London, Turner Street, London, UK

**Keywords:** oral cancer, screening, high risk, disadvantaged communities, Bangladeshi

## Abstract

**Background:**

UK oral cancer incidence has risen by 22% in the last 10 years. Oral cancer is often detected at a late stage when treatment is debilitating and the chances of survival are poor. Certain black and minority ethnic groups are at elevated risk of oral cancer due to the prevalence of risk factor behaviours. We describe the background to, the development of and outcomes of an oral cancer screening activity appropriate to the needs of members of a disadvantaged community at high risk of oral cancer, carried out between 2006 and 2008 in Tower Hamlets, East London.

**Methods:**

In all, 1320 people participated during 34 days of screening, divided into two phases (Phase I (2006/2007): *n*=485, Phase II (2008): *n*=835). Modifications to the delivery process were implemented for Phase II in an attempt to recruit more high-risk individuals and to improve screening specificity.

**Results:**

In total, 75 people were urgently referred for further investigation (Phase I: *n*=20, Phase II: *n*= 55). Nine were diagnosed with dysplastic lesions (Phase I: *n*=3, Phase II: *n*=6) and a further eight showed potentially malignant disorders without dysplasia (Phase I: *n*=1, Phase II: *n*=7). Screening participants with low levels of completed education (OR: 6.94, 95% CI: 1.66, 28.98) and who chewed paan with tobacco (OR: 8.01, 95% CI: 3.54, 18.08) were more likely to be referred for further investigation.

**Conclusion:**

The project offers insights for the further development of oral cancer screening interventions for disadvantaged communities.

Oral cancer is defined as cancers of the lip, tongue, oral cavity, oropharynx, hypopharynx and piriform sinus. The majority of tumours are squamous cell carcinomas, with the most common sites being the oral cavity (31%) and the tongue (29%). The least common site for oral cancer is the lip, accounting for only 6% of cases (Office for National Statistics, 1996–2005). UK oral cancer incidence rates have increased by 22% in the last 10 years (Office for National Statistics, 1996–2005). In 2005, 4900 people were diagnosed with oral cancer in this country (Office for National Statistics, 1996–2005). There are inequalities in oral cancer incidence and survival, with rates varying according to level of deprivation, geographical location and ethnicity (Office for National Statistics, 1996–2005; NCIN, 2009). The available data suggest that risk of oral cancer is higher among older Asian females than in females from the general population, although this is not the case for males (NCIN, 2009).

The average 5-year survival rate for cancer of the oral cavity is around 50% (Office for National Statistics, 1996–2005). This is because the majority of cases are diagnosed at a late stage, when treatment is debilitating and the chances of survival are poor (Murphy *et al*, 2007). Oral cancer survival varies considerably by stage at diagnosis and by site (Office for National Statistics, 1996–2005). Treatment for early stage oral cancer or oral dysplasia, tends to be considerably less invasive and debilitating (Lodi *et al*, 2006; van der Waal, 2009).

## The Bangladeshi community in Tower Hamlets

Oral cancer is undoubtedly an important public health issue, but as it is a relatively rare disease in the United Kingdom, targeted population strategies are likely to be most cost effective. From 2005 to 2008, Cancer Research UK ran an oral cancer project targeted at the Bangladeshi community in Tower Hamlets, East London, funded largely by the Chief Dental Officer, Department of Health.

Tower Hamlets is the third most deprived borough in England (Office for National Statistics, 2001). One third of the borough's population is Bangladeshi, totalling nearly 66 000. This is the largest Bangladeshi community outside of Bangladesh (Office for National Statistics, 2001). Conducting the project in this borough offered the opportunity to closely target a large community at high risk of oral cancer residing in a relatively small geographical area in a deprived part of London.

The Bangladeshi community exhibits a high prevalence of a number of oral cancer risk factors, including smoking, chewing tobacco and chewing areca nut but excluding alcohol consumption when compared with the general adult population (Croucher *et al*, 2003; Health Survey for England, 2004). Forty per cent of Bangladeshi men report being current smokers, compared to 24% of men from the general population. Although Bangladeshi women are much less likely to report being current smokers compared to women from the general population (2% compared to 23%), national estimates of chewing tobacco or areca nut use range from 16% of Bangladeshi women chewing paan with tobacco and 13% chewing paan without tobacco but with areca nut (Health Survey for England, 2004) to 48.5% of a sample of Bangladeshi women resident in Tower Hamlets chewing paan with tobacco (Croucher *et al*, 2003). As the Bangladeshi community of Tower Hamlets shows significantly different risk factor behaviours from the general UK population, highly targeted oral cancer screening is likely to be needed.

This paper aims to describe the development of an oral cancer screening activity appropriate to the needs of a disadvantaged community at high risk of oral cancer. Specifically, it will describe the delivery structure of the screening activity, explain the process followed and review and discuss some outcomes.

## Oral screening and potentially malignant disorders

There is significant potential for early detection of oral cancer because the oral cavity and oropharynx are relatively accessible and amenable to examination without invasive procedures. The existence of potentially malignant disorders of the oral mucosa implies there should be significant potential for prevention of oral cancer through oral visual screening (Scully and Porter, 2000).

Potentially malignant disorders are defined as ‘all clinical presentations that carry a risk of cancer’ (Warnakulasuriya, 2007). They tend to be linked to tobacco use (Napier and Speight, 2008). The proportion of potentially malignant disorders that transform into frank oral cancers varies greatly, based on characteristics of the disorder and its site, the patient's age and gender (Napier and Speight, 2008), and the patient's behaviour (Warnakulasuriya *et al*, 2008; van der Waal, 2009). Overall malignant transformation rate for white patches (leukoplakias) is estimated at around 1% per year (van der Waal, 2009) while it is thought that between 75% and 90% of red patches (erythroplakias) will undergo malignant transformation (Scully and Porter, 2000).

Dysplastic change is reported to be the best predictor of future malignancy, and the more severe the dysplasia, the greater the likelihood of malignant transformation (Schepman *et al*, 1998). Estimates suggest that around 50% of patients with dysplasia may go on to develop oral squamous cell carcinoma (Napier and Speight, 2008).

The transformation rate of other potentially malignant disorders of the oral and oropharyngeal mucosa is harder to quantify, ranging from 0.13% to 2.2% for all potentially malignant disorders combined (Napier and Speight, 2008). Of specific relevance to an Asian population, where areca nut (betel quid) usage is prevalent, is oral sub-mucous fibrosis (OSF). This potentially malignant disorder has a reported malignant transformation rate of 14% (Tilakaratne *et al*, 2006).

A number of possible screening techniques have been proposed for oral cancer. The simplest involves visual examination of the oral mucosa (Kujan *et al*, 2006). A recent Cochrane review concluded that there was not enough evidence to determine whether oral screening by visual examination, or any other modality, in the general population could reduce mortality from oral cancer (Kujan *et al*, 2006), but an increasing number of studies suggest that oral screening could feasibly be carried out cost effectively as part of routine dental inspection in NHS general dental practice (Field *et al*, 1995; Lim *et al*, 2003; Speight *et al*, 2006). This may be of limited relevance to the Bangladeshi population of Tower Hamlets who are known to have poor attendance rates at General Dental Practitioners (Pearson *et al*, 1999).

Cuba is one of the only countries in the world to report a national oral cancer screening programme. This programme uses annual visual examination in dental practices (Fernandez Garrote *et al*, 1995). While there is some evidence that repeated screenings led to a reduced likelihood of advanced stage oral cancer (Sankaranarayanan *et al*, 2002), overall there has been limited evidence of a shift from advanced to earlier stage oral cancer following introduction of the programme (Fernandez Garrote *et al*, 1995).

## Materials and methods

### Developing a delivery structure

The following steps were followed in developing a protocol for screening activity:

1. *Establishment of a community advisory group* This comprised local stakeholders from the Primary Care Trust Smoking Cessation and Dental Access teams, Cancer Services, Oral Medicine and Oral Maxillofacial Surgery teams, community organisations, as well as practice nurses and patient and community representatives. The group offered guidance on the development and promotion of oral cancer screening activity among the Bangladeshi community and the development of accompanying oral cancer awareness literature.
2. *Formal qualitative research* was conducted with the target population, by means of focus group discussions and key informant interviews, to inform development of the oral cancer screening activity and accompanying literature.
3. *Identification of screening sites* in the locality to undertake screening activity where a mobile dental unit provided by the Primary Care Trust could be used.
4. *Screening criteria established* Oral cancer screening involved visual examination of the soft tissue of the oral cavity and oropharynx, and palpation of the neck using standardised criteria. The activity was conducted by two registered dental practitioners after refresher training to ensure compliance with guidance (National Institute for Health and Clinical Excellence, 2005).
5. *Development of referral pathways* Patients could be referred directly from the mobile dental unit, either to the Department of Oral Medicine, Barts and the London Dental Institute for further investigation, and/or to local stop tobacco services for cessation support.
6. *Determination of inclusion criteria* In addition to being Bangladeshi or British Bangladeshi and a resident of Tower Hamlets, inclusion criteria for screening included being aged at least 30 years and practising one or more of the following health-compromising behaviours: smoking tobacco, chewing tobacco, chewing paan (betel quid) without tobacco. Potential participants falling outside these inclusion criteria were not actively recruited, but if they persistently sought screening, whatever their age, gender, ethnicity or behaviour, they were not excluded.

### The delivery process

Delivery of oral cancer screening in Tower Hamlets as part of this project was divided into two phases. *Phase I* involved 10 screening sessions, which took place in 2006 and 2007, and *Phase II* consisted of 24 screening sessions, which took place in 2008. Several refinements were made to the delivery process for Phase II.

In both phases, all screening sessions were held in the six wards within Tower Hamlets with the highest proportions of Bangladeshi residents based on ethnicity data from the 2001 census (Office for National Statistics, 2001). In Phase I, the 10 screening sessions were fairly evenly divided between these six wards, with exact locations of the mobile dental unit based on advice from the community advisory group. In Phase II, locations for conducting 24 oral cancer screening sessions were purposefully selected using ward ethnicity data from the 2001 census (Office for National Statistics, 2001), with the number of sessions conducted in each ward proportional to the size of the Bangladeshi population in that ward, with a higher number of screening sessions held in wards with the highest proportions of Bangladeshi residents. The exact location within each ward was again based on advice from our community advisory group.

Screening was undertaken using a mobile dental unit – a specifically adapted motor vehicle, housing a dental clinic with conventional reclining dental chair and telescopic high-intensity lighting. This provided a clean, safe and confidential environment for examination of the illuminated oral mucosa in the supine position with the availability of all the instruments and amenities that would be present in a permanent dental clinic. Use of the mobile dental unit addressed all as the issues arising from pre-trial attempts at mucosal examination using hand-held portable equipment, including inconsistent lighting intensity, confidentiality and modesty concerns when examining in busy areas, difficulty gaining adequate oral mucosal access without the patient in the supine position, concerns about the examiners' posture and lack of appropriate cross-infection control.

Ensuring the cultural acceptability of oral cancer screening activity was addressed by the use of bi-lingual (English/Sylheti) advocates. Again, the procedure was modified between Phase I and Phase II. In Phase I, the main roles of bilingual advocates were to provide language support during and after screening and to facilitate the referral process by following up referred patients individually. In Phase II, the advocates had a more integral role in the delivery process, continuing to provide language and other support during and after screening, recruiting participants to tobacco cessation, facilitating the referral process and collecting evaluation data and promoting oral cancer screening activity. As well as encouraging passing pedestrians to take part, this promotion of oral cancer screening involved placing oral cancer awareness and advertising health education material in areas where, from their local knowledge, the advocates knew that older Bangladeshis lived, or visited, such as community centres, shops and sheltered accommodation. In addition, at the referral stage, the advocates facilitated the distribution of referral letters, followed up the letters with telephone calls to ensure the letters had been received and understood, and offered to accompany patients to their referral appointment if required. This process ensured as many patients as possible complied with referral.

Final modifications to the delivery process for Phase II concerned selection, training and calibration of the dentists conducting screening. For Phase I, dentists with varying levels of experience in the diagnosis and management of oral cancer were recruited. They were standardised by attendance at a pre-screening refresher session where images of oral mucosal lesions were examined in relation to the NICE guidelines on the referral of suspected cancer (National Institute for Health and Clinical Excellence, 2005). A more robust standardisation and calibration process was developed for Phase II based on an updated protocol (Ikeda *et al*, 1995; Barnes *et al*, 2005). Just two dentists were recruited for Phase II, both with extensive clinical experience in diagnosing oral mucosal pathology at secondary referral centres.

### Assessing outcomes

The following outcomes of the activity are reported:

- numbers (Phases I and II) and demographic details (Phase II only) of screening participants and details of recruitment to local tobacco cessation services (Phase II only)
- numbers referred to secondary care services (Phases I and II)
- compliance with referral (Phases I and II)
- clinical outcomes and diagnoses (Phases I and II)
- socio-demographic and behavioural predictors of referral (Phase II only) using a retrospective case–control design, whereby participants referred were matched with multiple controls to increase study power
- financial considerations

During Phase II, a questionnaire was used to identify potential screening participants. Those identified as meeting the inclusion criteria were then asked to take part in a second interview. Information about age, gender, years of completed education, economic activity, self-reported health, oral cancer awareness and tobacco consumption was collected. The core content of the interview was provided by using validated questions taken from existing inventories (Humphris *et al*, 1999; Health Survey for England, 2004).

Data collection were approved by the local research ethics committee and analysed using STATA version 10. Participants gave their written consent to take part in the interview.

## Results

### Characteristics of screened population and recruitment to tobacco cessation

Between 2006 and 2008, a total of 34 screening sessions were undertaken in Tower Hamlets with 1320 individuals screened (485 in Phase I and 835 in Phase II). On average, 39 people were screened per session (range 19–82). Each session was ∼7 h in duration, undertaken between 0930 and 1630 with flexible break times dependent on the flow of patients.

The mean age of the screened population was 42.3 years (SD 15.9 years). As shown in Table 1, 45% of patients screened in Phase II were female, 84% were from the Bangladeshi community and 58% were tobacco or areca nut users. In Phase II, a total of 202 screening participants were recruited to tobacco cessation (i.e. 42% of participants who were tobacco users). Data collection in Phase I were incomplete.

### Referral, compliance and clinical outcomes

Of the 1320 individuals screened, 75 were referred for Oral Medicine consultation at Barts and The London Dental Institute. Table 2 shows the outcomes of these referrals. Despite attempts to ensure as many patients as possible complied with referral, 20 (27%) failed to attend either their initial hospital appointment or subsequent follow-up appointments such that a definitive diagnosis of their condition could not be made. Six out of 14 patients failed to comply with referral in Phase I (30%), while 14 out of 55 failed to comply in Phase II (25%). In all, 55 patients did attend after referral, and further investigations were instigated as appropriate to generate the diagnoses shown in Table 2.

None of those referred presented with frank oral cancer but 17 (31%) were diagnosed with potentially malignant disorders as shown in Table 2. Of these, nine had evidence of dysplasia (two moderate/severe) and five had OSF. The remaining 38 patients had benign lesions, of which the most common was keratosis (*n*=31).

Of the 55 patients attending the secondary referral service at Barts and the London, 35 (64%) remain under regular review including all those with potentially malignant disorders. These individuals exhibit a number of risk factors for the development of oral cancer and their pre-existing mucosal lesions indicate a significant risk of further lesions developing. Only 20 patients presenting with confirmed benign lesions and minimal risk factors, who had access to appropriate follow-up within primary dental care services, were able to be discharged from further review in the Department of Oral Medicine. In Phase I, 7 out of 14 (50%) of compliant referred patients remain under review, whereas in Phase II, 28 out of 41 compliant patients (68%) remain under review.

### Socio-demographic and behavioural predictors of referral

Data were available for 49 of the 55 patients referred in Phase II. They were matched with 344 non-referred controls. Features of the cases and controls are reported in Table 3. Screening participants with low levels of completed education (OR: 6.94, 95% CI: 1.66, 28.98) and who chewed paan with tobacco (OR: 8.01, 95% CI: 3.54, 18.08) were more likely to be referred to the secondary care service. For other characteristics – economic activity, general health, smoking cigarettes, bidi and chewing paan without tobacco – there were no statistically significant differences in the screened population compared with the referred population.

### Financial considerations

The 34 screening sessions in Tower Hamlets cost ∼£32 000 including all associated direct costs such as staff and hire of fully equipped mobile dental units but not including consultant time and hospital facilities for follow-up appointments. As 1320 people were screened during this period, the cost per screen was ∼£24, which is approximately half the figure for the NHS breast-screening programme that stands at £45.50 per screen.

## Discussion

This project has shown the feasibility of conducting oral cancer screening in a deprived borough in East London, using a mobile dental clinic with dental practitioners undertaking the screening, supported by ethnically matched advocates from the local community. In line with the aims of the project, over 90% of the screening attendees in Phase II were from the local Bangladeshi community. This project also confirms the importance of providing oral health services in community settings (Croucher and Sohanpal, 2006).

Of the 1320 people screened, 75 (5.7%) were referred to secondary care for further investigation. This is in line with previous UK oral cancer screening initiatives (Downer *et al*, 2006) and is almost identical to that observed in a large screening trial in Kerala, India (Sankaranarayanan *et al*, 2005). Those referred were more likely to be paan with tobacco chewers, confirming previous findings (Pearson *et al*, 1999; Jayalekshmi *et al*, 2009), and to have limited years of completed education.

Only 55 (73%) of the 75 referred to secondary care attended their appointment despite considerable efforts being made both by the secondary referral centre and the local advocates. This is clearly a concern. However, this attendance rate is comparable with that in other studies (Downer *et al*, 2006) and is somewhat higher than the 63% reported for the Kerala study (Sankaranarayanan *et al*, 2005). It is also worthy of note that in the national bowel cancer screening programme ∼20% of those with a positive faecal occult blood test do not attend for colonoscopy.

Reasons for non-attendance at secondary care were investigated by telephoning patients who initially failed to attend, but subsequently did so. Common reasons for initial non-attendance included language barriers, non-receipt of referral letter and a perceived difficulty in attending hospital. Among the 20 patients who never attended secondary care, it has been established that two had returned to Bangladesh but it was not possible to determine whether this was as a direct result of the screening outcome and associated psychological burden.

Among the 55 patients who attended secondary care, 17 (31%) were found to have potentially malignant disorders. Benign oral mucosal hyperkeratosis was by far the most common lesion, occurring in 31 (56%) of the 55 individuals. This is consistent with a paan chewing habit, where the rough fibres of the betel quid cause frictional damage to the epithelial surface of the mucosa (Lalli *et al*, 2008). In total, 35 (64%) of the 55 attendees at secondary care remain under follow-up because of their mucosal condition and associated risk factors. These can be considered as positive referrals.

One of the unique features of this project was the linkage between oral cancer and the tobacco cessation programme. In Phase II of the project, over 40% of tobacco users attending screening (202 of 485) were recruited to tobacco cessation.

### Future developments

This project has shown the feasibility and acceptability of oral cancer screening using a mobile dental unit among the Bangladeshi community of Tower Hamlets. It would now be valuable to test this approach in other high-risk communities.

Further investigation into the reasons for non-compliance with referral to secondary care is needed. Innovative use of the mobile dental unit could eradicate delay in obtaining a definitive diagnosis from initial screening if suspicious lesions were biopsied in the field. A brush biopsy and immediate cytological analysis could be an appropriately quick and minimally invasive procedure to undertake on the mobile dental unit. There would, however, be significant cost implications.

## Figures and Tables

**Table 1:** Details of screening participants in Phase II

	**Phase II (2008)**
Number of screening sessions	24
Number of individuals screened	835
Number of females screened (%)	375 (45%)
Number of Bangladeshis screened (%)	762 (91%)
Number of tobacco/areca nut users screened (%)	485 (58%)
Number of recruits to tobacco cessation	202

**Table 2:** Definitive diagnosis by secondary referral centre

**Clinical/histological**	**Number of patients (of those who attended oral medicine clinic)**		
**diagnosis**	**Phase I**	**Phase II**	**Total**
*Potentially malignant disorders*			
Dysplasia	3	6	9
Oral sub-mucous fibrosis	1	4	5
Lichen planus	0	3	3
Total potentially malignant disorders	4	13	17
			
*Benign lesions*			
Keratosis	9	22	31
Candidiasis	0	1	1
Fibro-epithelial polyp	0	1	1
Physiological pigmentation	1	2	3
Haemangioma	0	2	2
Total benign lesions	10	28	38
			
Total	14	41	55

**Table 3:** Predictors of referral

**Variables**	**Univariate analysis: OR (95% CI)**	***P*-value**	**Multivariate analysis: OR (95% CI)**	***P*-value**
*Years in education*				
19+	1.00		1.00	
15–18	2.81 (0.97–8.13)	0.04	1.98 (0.61–6.41)	0.25
Under 14	1.64 (0.57–4.69)	0.34	2.02 (0.62–6.57)	0.24
None	3.6(10.20–41.30)	0.01	6.94 (1.66–28.98)	0.01
Economically active	0.70 (0.29–1.68)	0.42	0.32 (0.09–1.15)	0.08
*Self-reported health*				
Good-excellent	1.11 (0.57–2.17)	0.73	1.60 (0.71–3.58)	0.25
*Self-reported oral health*				
Good-excellent	0.87 (0.39–1.94)	0.74	0.77 (0.33–1.78)	0.55
Any oral pain	0.98 (0.51–1.91)	0.97	0.86 (0.61–1.23)	0.43
Smoke cigarettes	0.81 (0.32–2.00)	0.65	0.64 (0.22–1.88)	0.42
Smoke bidi	1.58 (0.14–18.06)	0.70	1.84 (0.14–23.04)	0.63
Chew paan with tobacco	5.69 (2.66–12.18)	0.00	8.01 (3.54–18.08)	0.00
Chew paan without tobacco	0.56 (0.26–1.17)	0.12	0.73 (0.29–1.81)	0.50
OC awareness: 5+ correct	1.02 (0.52–2.04)	0.93	1.02 (0.53–2.45)	0.73

Results of univariate and multivariate conditional logistic analysis.
